# Looking through the Same Eyes? Do Teachers' Participation Ratings Match with Ratings of Students with Autism Spectrum Conditions in Mainstream Schools?

**DOI:** 10.1155/2012/656981

**Published:** 2012-05-08

**Authors:** Marita Falkmer, Richard Parsons, Mats Granlund

**Affiliations:** ^1^Department of Education, Municipality Council of Norrköping, Norrköping, Sweden; ^2^CHILD Programme, Swedish Institute of Disability Research and School of Education and Communication, Jönköping University, P.O. Box 1026, 551 11 Jönköping, Sweden; ^3^School of Occupational Therapy and Social Work and Curtin Health Innovation Research Institute, Faculty of Health Sciences, Curtin University, GPO Box U1987, Perth, WA 6845, Australia; ^4^CHILD Programme, Swedish Institute of Disability Research and School of Health Sciences, Jönköping University, P.O. Box 1026, 551 11 Jönköping, Sweden

## Abstract

To create an inclusive classroom and act accordingly, teachers' understanding of the experiences of participation of students with autism spectrum conditions (ASCs) is crucial. This understanding may depend on the teachers' professional experiences, support and personal interests. The aim of the present questionnaire study was to investigate how well the teachers' ratings of their students with ASCs' perception of participation matched with the students' own ratings. Furthermore, possible correlations between the accuracy of teachers' ratings and the teachers' self-reported professional experience, support (including support-staff), and personal interest were investigated. Teachers' ratings were also used to examine how their understandings correlated with classroom actions. The agreements between teachers' and students' ratings were moderate to high, and the ability to attune to the students' perception of participation was not affected by the presence of a support-staff. The teachers' personal interest in teaching students with ASC correlated with their accuracy, suggesting that this is a factor to consider when planning for successful placements in mainstream schools. Teachers' understandings of the students with ASCs' perception of being bullied or unpopular correlated with implementation of activities to improve the attitudes of classmates, but not with actions to enhance social relations for the students with ASC.

## 1. Introduction

Since the Salamanca declaration [1], taking its stand from the Convention on the Rights of the Child and the Convention on the Rights of Persons with Disabilities, inclusive schools have been the goal for many countries' school policies. If students with impairments are included in a mainstream school, they are afforded an opportunity to participate in activities and social interaction [2]. However, simply being in a mainstream school environment is not enough for participation to occur. Both environmental aspects, for example, attitudes of classmates and teachers, and personal characteristics of the student with impairment affect participation [3]. Participation per se is, however, not mentioned in the Salamanca declaration but the term is used in The Standard Rules on the Equalization of Opportunities for Persons with Disabilities [4].

Participation can be viewed as having two dimensions, namely, performing an activity and the perception of being involved in that activity. Actual, as well as perceived, availability and access to activities influence the performance dimension [3]. The perceived meaningfulness of the activity influences the perception of involvement and participation. Consequently, participation includes one aspect that can be observed and rated by others, that is, performing the activity, and a subjective aspect that is perceived and best rated by the individual, that is, the subjective feeling of involvement [5]. In order to evaluate the impact of inclusive school practices on students' participation within the school environment, both self-rated and observed participation are important outcome variables [6]. However, when teachers have been asked to rate how they think their typically developed students perceive participation in schools, the teachers' and students' ratings have been reported not to correlate [7]. This finding indicates that teachers focus on observed participation in schools but may lack insight into the students' experiences of participation. Attentiveness to the different aspects of participation is of importance for teachers since underestimation of participation-related problems perceived by the student is not uncommon [8]. In the worst case, discrepancies in teachers' and students' perception of the students' school situation could result in absent or delayed interventions.

To participate in school includes the possibility to partake and engage in activities within the physical, social, and academic school environment [9]. Teachers play a crucial role in the creation of an inclusive school setting, especially for students with impairments [10]. As barriers for inclusion and thereby participation in mainstream schools, teachers' lack of information about the individual student with impairments and insufficient education regarding the consequences of these impairments have been identified by included students and their parents [11]. Teachers' attitudes towards inclusion per se and their willingness to differentiate and individualise their classroom strategies are essential for an inclusive school [12]. From the students' perspective, a good interaction with the teacher influences their participation in a positive way [13] since the quality of the relationship between the teacher and the student with impairments also affects the relations between the student with impairments and his/her peers [14].

Being able to collaborate with special educators and specialised support-staff has been shown to positively affect teachers' attitudes towards inclusion [12] since it seems to enhance their confidence in executing flexibility in their teaching [12, 15]. There are, however, inconclusive findings on how the presence of support-staff in the classroom affects the relationship between students with impairments and their teachers [16–18]. Earlier findings suggesting that the teachers tend to withdraw and leave most of the interaction with the student with impairments to the support-staff [16, 17] have not been confirmed in a recent study [18]. This may be due to the fact that the role of a full-time individual support-staff in the classroom is complicated [16, 19]. A support-staff can be of great help for the student and the teacher, but a too close presence of an adult can exclude the student with impairments from interactions with classmates. Furthermore, if the support-staff is the one primarily responsible for instructions and adaptations of learning material and curriculum, the student's participation in academic activities and learning may also be hampered. Previous studies suggest that assigning a support-staff to the classroom may be more beneficial for student participation than assigning him/her to an particular student [19] since the teachers tend to keep the primary responsibility for planning and instructing students with impairments to a greater extent when the support-staff is assigned to the whole class [19]. To enhance participation, both support-staff and the teacher need adequate training and sharing the responsibility for the student with impairments [18]. Furthermore, a close collaboration between the teacher and the support-staff, in regard to planning and implementing strategies for the student to achieve both academic and social skills goals, is essential [18].

Lack of social competence, for example, difficulties in understanding social information, restricted capacity to adapt the way emotions are expressed and to understand and respond to expressions of emotions from others, is a personal characteristic that is likely to exclude students from participation in mainstream schools [20, 21]. This characteristic may be particularly pronounced in students with autism spectrum conditions (ASCs). The term ASC covers several diagnoses, for example, autism and Asperger syndrome. ASC are characterised by communication difficulties and impairments in social competence and imagination [22–24]. Difficulties with social imagination lead to problems with flexibility and an impaired ability to anticipate consequences of one's actions in different situations [24]. Since the manifestations of ASC symptoms vary between individuals and across ages depending on the environment, the needs of this group of students may be particularly difficult to understand for mainstream school professionals [24]. While teachers in mainstream school generally perceive that they have a positive relation with their students with ASC [18], specific student characteristics, such as difficulties in understanding social emotions, motivation, communication, and adapting behaviour among the students, constitute factors that negatively affect these relations [18, 25]. In fact, students with ASC in mainstream schools are identified as being at risk of dropping out of [26] or being excluded from mainstream schools [27]. The reason is mainly not because of intellectual problems, but rather due to a nonadaptive education style and poor reception by teachers and peers, leading to social alienation and lack of participation in a, from the student's perspective, meaningful school context [26].

There are limited evaluations on how to best enhance the development of social skills in children with ASC [28]. Inclusion in mainstream schools provides students with ASC with access to socially competent peers, which is a crucial prerequisite for social learning [28, 29]. Students with ASC in mainstream schools show higher levels of social interaction and have a larger social network compared with students in segregated school settings [2]. However, inclusion on its own may only provide the students with ASC with an opportunity to establish friendships [30] and result in a meek, but not significant, increase of social interactions [28]. To be included in a group of socially competent peers is thus necessary but not sufficient enough for students with ASC in order to develop social skills [28, 29]. Social skills interventions are necessary [28, 29]. A range of social skills interventions seem to have positive effects on children with ASCs' development of social initiation and responses, as well as on social problem solving and play skills [2]. Interventions aimed at enhancing social skills have the best results when directed not only to the child with ASC, but also to his/her classmates [28, 29]. Consequently, such interventions should target both. Social skills interventions through teacher-facilitated activities, both in the classroom and during recess to encourage interactions between students with and without impairments have been proven to be important aspects of teaching in mainstream schools [31].

Previous research suggests that for most students with ASC there is a need to continuously include activities aimed at developing social interaction in their educational environment [28]. However, the student's communication difficulties can result in teachers not being aware of the need to do so, since many students with ASC who perceive their mainstream school situation as difficult and exclusive do not tell their teacher, or even their parents [24]. As an illustration, teachers have been found to rate the social interaction of students with ASC higher than the students themselves [32]. Positive teacher/student interaction is therefore not only imperative to understand the needs of students with ASC. It is also a means to adapt classroom strategies in an inclusive way [24] and a source that can facilitate planning and execution of interventions that support an accepting social and attitudinal climate in the classroom when needed [18, 33].

To conclude, teachers need to be able to take their student's perspective in a genuinely empathetic way and have a thorough understanding of that individual student, in order to create an inclusive school situation [24]. This implies that in the school environment, the teachers take great responsibility in observing the level of participation of each student with ASC [24] and use this information, in order to understand the student's situation. An indication of the teachers' ability to take their student's perspective is a mutual understanding of the student's degree of participation in school, measured as the agreement between teachers' and their students with ASCs' rated participation [3]. However, research in regard to teachers' accuracy in assessing these students' participation is limited [8]. Thus, the overall aim of the present study was to investigate how well the teachers' ratings of how they thought the students with ASCs' would rate their participation in mainstream schools matched with the students' own rating of his/her perceived participation. The research questions were the following.

- How well do the teachers' ratings of their student's perception of participation match with the students with ASCs' own ratings?
- How does the presence of support-staff affect the teachers' rating accuracy?
- How does the accuracy of teachers' ratings correlate with teachers' self-reported professional experience, perceived support and personal interests?
- How does teachers' understanding, reflected by their ratings of the students' perceived level of participation, influence their actions in the classroom?

## 2. Method

### 2.1. Participants

Through The Swedish National Autism and Asperger Association, their local associates, local paediatric clinics, local support team in special education, and personal contacts, information letters and interest announcements were sent out to families included on their registers having a child in year 3–6 in a Swedish mainstream school. Parents who consented to let their child participate in the study contacted the first author. The child was included in the study if the parents, the child, the headmaster of the school, and the classroom teacher/teachers, in that particular order, all gave their written informed consent/assent.

As shown in Table 1, 22 students with ASC were included in the study (16 boys), aged 9–13. Two of the students attended the same class and, consequently, 21 different classes were included. Inclusion criteria for the students were that the parents reported that their child had an ASC diagnosis set by a paediatric clinic. The diagnosis was confirmed by the school and known to the teachers. The participating mainstream school students were all reported to follow the national curriculum, which implies that they did not have an intellectual impairment.

Since some classrooms had more than one teacher working regularly with the students, only the teacher with the main responsibility for the student with ASC was included in the analysis. As shown in Table 1, the study included 21 teachers (16 women). However, one teacher provided ratings for two different students. The reported teaching experience of the participating teachers ranged between 4 and 35 years and their experience of working with students with ASC ranged from 1 to 6 years (Table 1). While all participating teachers had a formal teacher training, none of them had a formal special needs teacher education. On one statement in the questionnaire “*I have got education specifically on ASC*,” the teachers responded on a five-step response scale ranging from “*not at all true*” to “*completely true*.” Fifteen teachers (71%) responded on the two lowest scale steps. Only three teachers reported that it was “*completely true*” that they received such training (Table 1). The communities were small to medium sized with respect to number of inhabitants [34], with no metropolitan communities included in the present study.

### 2.2. Participation Questionnaire

Data on perceived participation were collected through a questionnaire regarding students' self-rated participation. The questionnaire was an adapted version of autonomy, locus of control, and participation scales from a previously used set of questionnaires (children's participation in school (age 7–12) and Adolescents' participation in school age 13–17) aimed to measure aspects of perceived participation [5, 13, 35]. The original questionnaire consisted of a Swedish version of an availability and participation scale [9] and of a Swedish version of autonomy and locus of control scales from The Arc's self-determination scale [36], adapted to school age children and teenagers. When used with Swedish children with impairments, moderate to high internal consistency and construct validity have been reported for the questionnaire packages (children's participation in school and adolescents' participation in school) [5, 13, 35, 37]. The adaptation consisted of creating a merged version by selecting statements from the two questionnaires. Since self-determination has been shown to contribute to students' participation in mainstream schools (12), nine statements aimed to capture the autonomy of the students by asking, for example, “*I want to help my classmates in school*” and “*I want to participate in physical education (P.E.)*,” were added to the questionnaire. In addition to demographic information regarding age, sex, and school year, the questionnaire used in the present study comprised 46 statements (presented in Table 5).

- Six **do** statements, for example, “*I meet my classmates after school*,” asking the student/teacher to indicate what was most in accordance with what the student usually would do, choosing from 4 given alternatives: “*I never do that even if I have the possibility*,” “*I do that sometimes, if I have the possibility*,” “*I do that most of the times if I have a possibility*,” “*I always do that if I have a possibility*.”
- Thirteen **agree** statements, for example, “*It is hard for me to get friends*,” asking the student/teacher to indicate to what degree he/she agreed with the statement being true for the student's situation, choosing from 4 given alternatives, “*never true*,” “*sometimes true*,” “*often true*,” “*always true*.”
- Twenty-seven **frequency** statements, some of them asking the student/teacher to rate the frequency, for example, “*I participate in P.E.*,” and some of them phrased in order to rate the level of self-determination in regard to these activities, for example, “*I want to participate in P.E.*”. The ratings were done on a 5-point graded scale: “*almost never*,” “*infrequently*,” “*neither seldom, nor often*,” “*frequently*,” “*almost always*.”

The participation questionnaires were handed out to the students during lecture time and collected by the first author who was present on site. Assistance and explanations were offered to students struggling with their reading.

At the same time, the participation questionnaire was distributed to the classroom teachers. It was modified so that it asked the teachers to rate how they thought that their student(s) with ASC would rate his/her level of participation on each of the 46 statements.

Two sets of statements were used to test the intrarater reliability of the participation questionnaire. The first set, “*I want to be with classmates during recess/I want to be alone during recess*,” had a four-step Likert response scale. The second set, “*It is easy for me to get friends/It is hard for me to get friends*,” had a five-step Likert response scale.

### 2.3. Teacher Questionnaire

A second questionnaire that consisted of 54 questions and statements was created, in order to obtain information regarding factors that were supposed to influence the teachers' relation with students with ASC. It also captured self-reported classroom activities enhancing participation. The questionnaire contained two questions regarding teaching experience:  “*I have worked as a teacher for … years*” and “*I have worked with students with ASC for … years*,” one statement regarding ASC-specific education, that is, “*I have been trained to work with students with ASC*,” rated on a five-step Likert response scale graded from “*not at all true*” to “*completely true*” and questions regarding support from and collaboration with teacher assistants, special education teachers, or otherwise specialised staff. The questionnaire also included statements regarding the degree of adaptations the teacher had made, for example, “*I always have to adapt tasks to the student with ASC's abilities when he/she studies in the classroom*.” These statements werealsorated on a five-step Likert scale graded from “*not at all true*” to “*completely true*.” In addition, the questionnaire included frequency statements to assess how often the teacher executed activities aimed at improving the attitudes and social relations in the classroom, for example, “*I do specific activities to enhance social relations for the student with ASC*.” These statements were graded on a 5-step response scale, namely, “*every week*,” “*every month*,” “*a few times every semester*,” “*a few times every school year*,” and “*never*.” There was also an option to report another interval. Additionally, there were open-ended questions, in which the teachers were asked to provide examples of the kind of activities they usually implemented and materials they used.

One set of statements,* “I always have to adapt tasks to the student with ASC's abilities when he/she studies in the classroom*” and “*I never have to adapt tasks to the student with ASC's abilities when he/she studies in the classroom*,” with a five-step Likert response scale was used to test the intrarater reliability of the teacher questionnaire.

### 2.4. Statistical Analyses

Four aspects of agreement were used as outcome variables in the analyses. Teachers' and students' ratings were recorded as “total agreement,” “near agreement,” and “disagreement.” Since “near agreement” indicated only a one step discrepancy between the teachers' and the students' ratings on either the four- or the five-step scales, the sum of “total agreement” and “near agreement” was also used as a variable, indicating a reasonable level of agreement.

The intrarater reliability of the questionnaires was tested with Cronbach's Alpha. Variables on interval and ordinal scales were tested for normal distribution with the use of Kolmogorov-Smirnov test. Mann-Whitney *U* tests and Spearman's correlations were calculated, with the *α*-level set at. 05. A power calculation indicated that the 22 pairs of student/teacher questionnaires would be adequate to identify a correlation coefficient of 0.44 or stronger with *β*-level = .2 (power = 80%) and the *α*-level set at  .05. Weighted Kappa analyses on student/teacher agreements were calculated where appropriate.

Teacher accuracy (calculated as the sum of total agreement and near agreement) in rating perceived participation in their students with ASC was created as a variable representing the teachers' understanding of the student with ASCs' participation. This variable was used for multiple correlation analyses with the teachers' self-reported professional experiences, perceived support, and personal interests, in addition to planned activities (Table 4).

Furthermore, since it was of interest to examine whether teachers' ratings influenced their actions in the classroom or not, teachers' ratings on selected statements from the participation questionnaire and their self-reported frequencies of classroom activities were used for two sets of correlation analyses. Firstly, teachers' ratings on three statements from the participation questionnaire, “*I am bullied at school*,” “*It is hard for me to get friends*,” and “*My classmates like me*,” rated as 1: “*not at all true*” –5: “*completely true*,” were all tested for possible correlations with their ratings on two statements from the teacher questionnaire, namely, “*I do specific activities to improve the attitudes of classmates towards the student with ASC*” and “*I do specific activities to enhance social relations for the student with ASC*,” rated as 1: “*every week*” *–*5: “*never*.” These statements from the participation questionnaire were chosen since if rated negatively they could be considered as reasons for teachers to implement activities aimed at enhancing social relations and/or activities to improve the attitudinal climate in the classroom. Secondly, teachers' ratings on the statement “*I can understand when my teacher explains to the class what to do*,” rated as 1: *not true* −5: *completely true*, in the participation questionnaire, and their ratings on the statement “*I always have to adapt tasks to the student with ASC's abilities when he/she studies in the classroom*,” rated as 1: *not true* −5:* completely true*, from the teacher questionnaire were tested for possible correlations.

### 2.5. Ethical Considerations

All the participants with ASC required their parents' written informed consent and their own written assent, prior to participation in the study. They were all given information on the aim and the procedures of the study and how data would be collected, stored confidentially, and published. On every occasion the students and the teachers were reminded verbally and in writing that they could choose to quit or not to answer some or all of the statements at any time and without any explanation required. The study design and procedures conformed to the Helsinki declaration, and the project was approved by the Regional Ethical Committee in Linköping, Sweden (Dnr 175-08).

## 3. Results

As shown in Table 2, the intrarater reliability coefficients of the questionnaires ranged between  .74 and  .95.

When including all the 46 statements, the mean percentage of total agreements between students and teachers was 33% (SD 9%) and the mean near agreements was 41% (SD 11%). When the total and near agreements were added together the mean was 74% (SD 14%). The distribution of total teacher/student agreement, near agreement, and disagreement, is presented in Table 5.

When comparing the mean percentage of agreements, near agreements, and disagreements and the sum of agreements and near agreements between teachers that worked alone in the classroom (*n* = 9) and teachers that worked together with a full-time support-staff/other teacher (*n* = 12) no differences were found.

To allow for a detailed examination of on which statements the disagreements were more or less frequent, all 46 statements in the questionnaire were analysed separately. Two sets were formed using the “10% or less disagreement” and “40% or more disagreement” cut-off values. As shown in Table 3, most of the statements in which there was more than 40% disagreement seemed to be related to aspects on, or perceptions of, social interactions.

**Figure 1:**
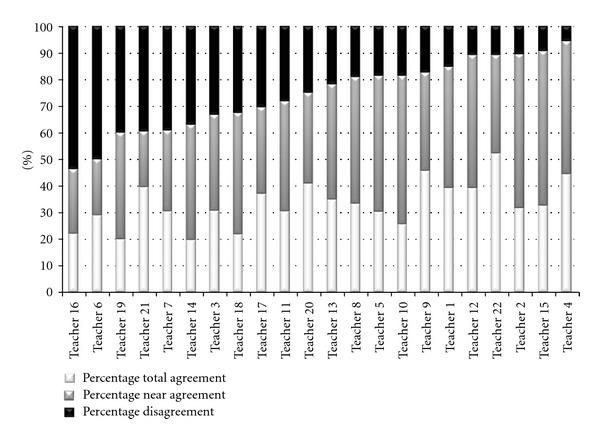
The distributions of total agreement, near agreement, and disagreement for each teacher. Teachers labelled 14 and 15 are the same teacher making two ratings in relation to the two different students with ASC in the same class.

The distributions of total agreements, near agreements, and disagreements for each teacher are shown in Figure 1.

Based on the outcome of the sum of total agreement and near agreement (Figure 1), that is, the teachers' accuracy, correlation analyses with teachers' self-reported teacher experiences, perceived support, planned activities and personal interests were performed. As shown in Table 4, the only significant correlation was a positive association between the degree of choosing to teach students with ASC and teacher accuracy.

A significant correlation was found between the teachers' ratings on “*My classmates like me*” and their reported frequency on the statement “*I do specific activities to improve the attitudes of classmates towards the student with ASC*” (rho  .51, *P* = .03), indicating that if the teacher understood it as if the student with ASC perceived that he/she was not liked, activities to improve attitudes of classmates were more often implemented. Another correlation was found between teachers' ratings on “*I am bullied at school*” and their reported frequency on the statement “*I do specific activities to improve the attitudes of classmates towards the student with ASC*” (rho −.51, *P* = .03), indicating that if the teacher understood it as if the student with ASC perceived him/her-self as being bullied, activities to improve attitudes of the classmates were implemented. 

Yet another significant correlation was found between teachers' ratings on “*I can understand when my teacher explains to the class what to do*” and their reported frequency on the statement “*I always have to adapt tasks to the student with ASC's abilities when he/she studies in the classroom*” (rho −.53, *P* = .02), indicating that if the teacher understood it as if the student did not understand, the teacher more frequently adapted the task*.*

**Figure 2:**
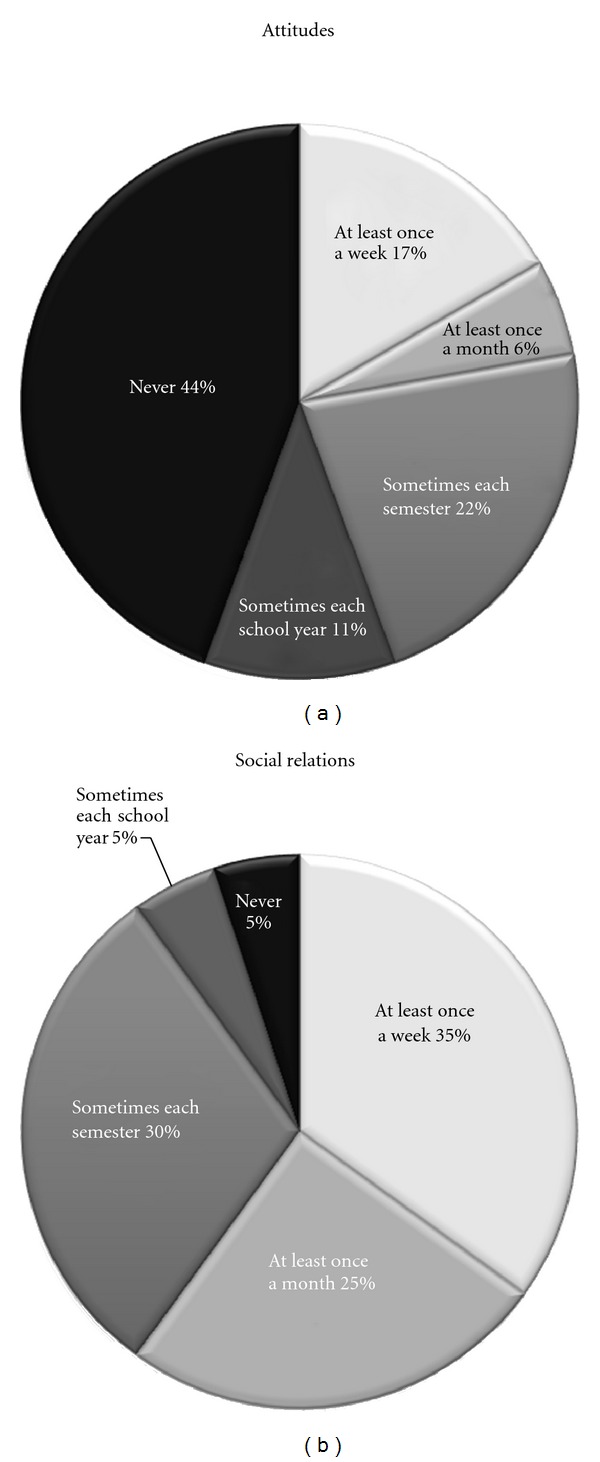
(a) Teachers' (*n* = 20) reported frequency of activities aiming to “*… improve the attitudes of peers towards the student with ASC*.” (b) Teachers' (*n* = 20) reported frequency of activities aiming “*… to enhance social relations of the student with ASC*.”

As shown in Figure 2 the results from the statements, in which the teachers rated the frequency of activities executed in the classroom, showed that at least once a week 35% of the teachers implemented activities aimed “*… to enhance social relations of the student with ASC*” and 17% “*… activities to improve the attitudes of classmates towards the student with ASC*.” 

In the open-ended question where teachers could provide examples of the activities carried out, in order to achieve the specific aim of enhancing social relations for the student with ASC, 17 teachers answered by giving examples, for example, “*I do activities where the students can practice collaboration, to work in groups and we try to do fun things together*.” Six of the examples named specific materials developed especially for the use of developing social relations in classrooms, aimed at teaching emotional intelligence (EQ), communication, and problem solving skills (http://www.projektcharlie.se/ny_sida_1.htm). Two teachers used strategies specially developed for students with ASC “*I do Social Stories when there is a need to*,” and “*I sometimes do Comic Strip Conversations*.” 

On the second open-ended question where teachers could provide examples of the activities they carried out, in order to improve the attitudes of classmates towards the student with ASC, 19 teachers answered by giving examples, for example, “*We do activities where we discuss values, practice collaboration and we play games*” and “*In the classroom, we often talk about the fact that we all are unique and different, but that we most of all have a lot in common, after all*”. Five of the examples referred to the use of material or programs developed especially for the purpose of improving attitudes in peer groups. No teacher mentioned strategies specially developed for students with ASC. 

## 4. Discussion

The questionnaire format was seen as providing students with ASC an opportunity to reflect on and give information about their perceived school situation since students with ASC tend to be more comfortable with visual than with verbal information [38]. However, all questionnaires are subject to interpretations by the respondents, and the way to control it is to test the intrarater reliability. The high intrarater reliability scores suggest that students with ASC and their teachers were consistent in their answers, which indicates that they understood the statements in a satisfactory way. 

The overall total agreement between teachers' and students' ratings regarding the students' perceived participation was generally moderate. However, when considering the combined measurement of total agreement/near agreement, teachers and students showed good agreement. This finding is encouraging, given that it is interpreted as a measurement of the teachers' insight into, and awareness of, the students' perception of their participation in mainstream schools. The results contrast a Swedish report, in which no correlations were found between typically developed students' and teachers' ratings of participation [7] and may indicate that teachers do pay extra attention to students with ASC. 

Interaction is an important way to gain insight into the students' situation. Previous studies [16, 17] have suggested that teachers who have a support-staff in the classroom tend to have limited interaction with the student with special needs. It could be presumed that the ability to attune to the students' perception of participation also could be affected by the presence of a support-staff. The present study could not confirm this. Despite not explicitly examining interactions between teachers and students, the high agreements between teachers' and students' ratings could be interpreted as being more in line with other findings indicating that the presence of a support-staff does not necessarily affect teacher/student relations negatively [18]. 

Furthermore, the results indicate that it is somewhat harder for teachers to rate in accordance with their students on aspects of participation that are not obviously related to the students' activities. For example, there was a 42% disagreement on the statement “*I am bullied at school*.” Previous studies have reported that most students with ASC in mainstream schools have experienced bullying and that there is a lack of awareness about this among school staff [8, 15, 39]. Hence, teachers need to be observant when it comes to the occurrence of bullying of students with ASC, since awareness is one prerequisite for taking actions. The use of tools that allow the students to continuously rate their own perceptions of their participation should be implemented in mainstream schools. Such self-ratings are likely to be useful to evaluate the inclusiveness of mainstream schools and the effectiveness of implemented inclusive strategies. 

An interesting observation is that the teacher who rated two students with ASC in the same classroom had a 62% sum of agreement and near agreement on the ratings of one student and 90% with the other. This finding is in accordance with previous research indicating that the individual characteristics of the student are important factors that strongly influence the relation between a teacher and a student with ASC [18]. 

The fact that no significant correlations were found between teachers' education in ASC, collaboration with special educators, or other support-staff and teachers' accuracy on students' ratings is most likely a result of the small number of teachers and students with ASC in the present study, since the importance of these factors for an inclusive mainstream school has been reported in elsewhere [11, 12, 40]. A limited possibility for teachers to make a choice to work with students with ASC became evident since only four teachers reported some level of personal choice. The positive correlation between the degree of personal interest to teach students with ASC and participation rating accuracy suggests that teachers' personal interest is a factor to take into consideration when planning for a successful placement of a student with ASC in mainstream schools. However, these results should be interpreted with caution due to the limited amount of data, and more research on factors that enhance teachers' insight in their students with ASC's perception of participation in mainstream schools is needed. 

The teachers reported a higher frequency of implementing activities aimed to enhance social interactions than of activities improving attitudes. However, significant correlations were only found between the statements relating to bullying and the perceived popularity of the student with ASC, and activities to improve attitudes. This finding indicates that teachers who understood that their student with ASC perceived him/herself as being bullied did not report a corresponding high frequency of implementing activities aimed at social relations. Instead, they more commonly implemented activities to improve the attitudes in the peer group. Although not correlated with whether their student was perceived as bullied or not, one-third of the teachers did report implementing activities aimed at enhancing social relationship on a weekly basis. The high frequency may be explained by the teachers interpreting any group activities the students do as enhancing social relations. However, for many students with ASC that is not enough. To simply be included in any random social activity will not automatically enhance social learning. There is a need to continuously implement activities with individualised social relation goals, in order to develop their social relation skills [28]. Hence, activities aimed at enhancing social participation should be seen as an integral part of the curriculum, especially when students with ASC are included in mainstream schools. The importance of individualised, teacher-planned and facilitated social activities, both in the classroom and during recess, may also have to be further stressed in teacher training. 

The lack of examples, both regarding attitudinal improvements and enhanced social interaction where teachers used material especially developed for students with ASC, could imply that more knowledge on, and usage of, such material is needed in mainstream schools. The lack of exemplified teaching material also indicates that in teacher training and continuing courses such materials should be introduced. 

A limitation of the present study is that the children's diagnosis was not specified and the ASC was not confirmed by medical records but by parents and schools. Furthermore, possible intellectual impairments were not checked for. However, the participating students did attend mainstream schools and were reported to follow the national curriculum, which, as mentioned, implies that the students did not have an additional intellectual impairment. The small number of teachers and students with ASC in this study implies that the results should be viewed with caution. The selection bias is also a factor to consider. Unfortunately, it is not easy to assess in which direction the bias may have affected the results. 

The correlation analyses in the present study suffered from type II errors, since a correlation coefficient of  .44 was the lower boundary detectable with a power of 80%. This means that several of the correlation analyses related to the teacher questionnaire may, in fact, have been significant although the present study could not detect it. However, correlation coefficients weaker than.44, which means an explained variance (*r* ^2^) of 19%, are usually of low or no relevance.

## 5. Conclusions

The overall agreement between teachers' and students' ratings regarding the students' perceived participation were moderate to high. The teachers' ability to attune to the students' perception of participation was not affected by the presence of a support-staff. However, the relatively high percentage of disagreement regarding the perception of being bullied stresses the need for teachers to be observant when it comes to the occurrence of bullying of students with ASC. The use of tools that allow the students to rate their perceptions of participation could, if implemented in mainstream schools, be useful to evaluate the inclusiveness of mainstream schools and the effectiveness of implemented inclusive strategies. Correlation analyses indicate that teachers' personal interest is a factor to take into consideration when planning for a successful placement of a student with ASC in mainstream schools. Correlation analyses also indicated that teachers did implement activities to improve the attitudes of classmates if the student with ASC was bullied or unpopular. Furthermore, teachers adapted tasks to the student with ASC's abilities to enhance their understanding. However, teachers' understanding of the students' participation did not correlate with taking actions to enhance social relations for the students with ASC. The importance of individualised, teacher planned and facilitated social activities, and knowledge of material and programs especially developed for enhancing social participation in students with ASC, may have to be further stressed in mainstream schools and in teacher training. 

## Figures and Tables

**Table 1:** Demographic information about the participating teachers, students, and the municipalities the mainstream schools were situated in. Standard deviation: SD, Male: M, female: F, and not applicable: N.A.

Teacher number^‡^	Sex (teacher)	General teaching experience (years)	Experience of teaching students with ASC (years)	Full-time support-staff	Education about ASC^§^	Degree of choice^†^	Total number of students in the class	Number of students with ASC in class	Sex (student with ASC)	Age (students with ASC)	Grade	Number of community inhabitants
1	M	10	3	Yes	2	3	16	1	F	11	5	135,500
2	F	14	3	Yes	5	3	18	1	F	9	3	4,500
3	M	32	1	Yes	2	1	18	1	M	11	4	18 000
4	F	10	1	Yes	2	2	16	1	M	10	4	10,100
5	F	35	6	Yes	5	1	30	1	M	12	6	38,600
6	M	16	—^¥^	No	2	1	20	1	M	11	5	26,000
7	M	12	3	Yes	3	1	15	1	M	12	6	12,400
8	M	8	1	Yes	2	2	22	1	M	10	4	61,300
9	F	13	1	Yes	2	1	18	1	F	9	3	2,200
10	F	8	3	No	5	1	21	1	M	11	5	27,100
11	F	12	1	No	2	1	16	1	F	11	4	24,000
12	F	12	—^¥^	Yes	1	1	21	1	M	12	6	5,400
13	M	6	5	No	2	1	17	1	M	12	5	18,000
14*	F	4	1	No	2	1	16	2	M	10	4	96,300
15*	F	4	1	No	2	1	16	2	F	10	4	96,300
16	F	9	4	No	2	1	25	1	F	10	4	16,800
17	F	4	1	Yes	3	1	17	1	M	10	4	32,400
18	F	12	3	Yes	2	1	21	1	M	11	5	55,500
19	F	22	5	No	2	1	11	1	M	10	3	33,800
20	F	12	2	Yes	1	1	29	1	M	12	6	23,400
21	F	—^¥^	—^¥^	Yes	—^¥^	—^¥^	20	1	M	13	6	130,100
22	F	14	6	No	2	1	17	1	M	12	5	18,000

Mean	N.A.	13.2	2.6	N.A.	2.0	1.3	17	N.A.	N.A.	10.7	5	N.A.
SD	N.A.	8.0	2.0	N.A.	1.2	0.6	4.5	N.A.	N.A.	1.1	1.0	N.A.
Median	N.A.	12.0	3.0	N.A.	2	1.0	18	N.A.	N.A.	11	4.5	N.A.

^‡^ In accordance with Figure 1.

*Teachers nos. 14 and 15 are the same person but she rated two different students and, hence, in the results her ratings are presented as two teachers' ratings.

^¥^Data missing.

^§^ Statement in the teacher questionnaire formulated as *“I have got education about ASC”* (1:* “not at all true” –*5:* “completely true”*).

^†^ Statement in the teacher questionnaire formulated as *“I have chosen to work with students with ASC”* (1:* “not at all true” –*5:* “completely true”*).

**Table 2:** Cronbach's alpha values of intrarater reliability in the two questionnaires, across teachers and students and across 4- and 5-step Likert scales.

Participation questionnaire	Teachers	Students with ASC
4-step Likert scale	.83	.87
5-step Likert scale	.95	.85

Teacher questionnaire	.74	
5-step Likert scale		

**Table 3:** The statements on which the students with ASC and the teachers disagreed the most and the least, presented with Weighted Kappa values*, means^1^, standard deviations (SD), medians, and interquartile ranges (IQRs). Not applicable: N.A.

Statements	Disagreement	Weighted Kappa*	Scores (mean^1^ (SD) and median (IQR))	
	≥40%		Students with ASC	Teachers

*Me and my classmates are together as long as I want to*	40.0	0.03	3.3 (1.3), 3.0 (1.0)	N.A., 4.0, (2.0)
*I want to help my classmates in school*	40.0	0.13	3.5 (1.4), 4.0 (2.0)	3.4 (1.0), 3.0 (1.0)
*I talk to teachers during recess*	40.0	>0.01	4.0 (1.2), 4.0 (2.0)	N.A., 5.0 (1.0)
*I am bullied at school*	42.1	N.A.	2.5 (1.3), 2.0 (2.0)	N.A., 1.5 (2.0)
*I work with different learning materials than my classmates*	42.9	N.A.	2.6 (1.3), 3.0 (2.0)	N.A., 1.0 (1.0)
*I can like a friend even if we sometimes disagree*	57.1	0.25	2.8 (1.1), 3.0 (2.0)	N.A., 3.0 (1.0)

	≤ 10%		Students with ASC	Teachers

*I go on outings when the school arranges it*	4.8	N.A.	N.A., 5.0 (0.5)	N.A., 5.0 (0.0)
*My teacher helps me to concentrate during lessons*	5.0	N.A.	3.5 (1.2), 4.0 (1.0)	3.7 (1.0), 4.0 (1.0)
*I want to participate in P.E. *	5.3	0.64	3.9 (1.6), 4.5 (1.8)	N.A., 5.0 (1.0)
*I talk to my classmates on the phone/Internet after school*	5.5	N.A.	2.0 (1.0), 2.0 (2.0)	N.A., 1.0 (1.0)
*It is hard for me to get friends*	5.9	N.A.	2.1 (0.9), 2.0 (0.8)	2.7 (0.8), 2.5 (1.0)
*I meet my classmates after school*	9.1	0.13	2.1 (0.9), 2.0 (1.3)	N.A., 2.0, (0.0)
*I participate in P.E.*	9.5	0.51	N.A., 5.0 (1.0)	N.A., 5.0 (0.3)
*I want to go on outings when the school arrange it*	10.0	N.A.	N.A., 5.0 (2.0)	N.A., 5.0 (1.0)

^1^ Mean values are presented where applicable depending on distribution of the data.

*Weighted Kappa value was not applicable when the answers were not symmetrically distributed.

**Table 4:** Correlation analyses between agreement (measured as the sum of total agreement and near agreement) and teachers' self-reported professional experiences, perceived support, personal interests, and planned activities. Ratings were done on a 5-step Likert scale unless otherwise indicated. *Indicates a significant correlation, and *n* indicates the number of respondents.

Item (number of correlated responses in brackets)	Rho value	*P*-value
Number of years as teacher (*n* = 20) (in years)	.004	.99
Number of years working with students with ASC (*n* = 18) (in years)	.003	.92
*Degree of choice in working with students with ASC *(*n* = 20)	*.54*	.001*
Training provided on ASC (*n* = 20)	.009	.97
Information from the student's parents about the student (*n* = 20)	−.006	.98
Special education teacher works separately with the student with ASC (*n* = 20)	−.106	.66
School psychologist works separately with the student with ASC (*n* = 20)	−.02	.93
Support-staff works separately with the student with ASC (*n* = 19)	−.22	.36
Active participation of the school's support team in the planning for the student with ASC (*n* = 20)	.37	.11
I experience that the student gets the support he/she wants to have (*n* = 20)	.19	.43
I experience that the student gets the support he/she needs (*n* = 20)	.03	.91
I experience that I get the support I need (*n* = 20)	−.14	.57
Percentage of time that student has a personal assistant in the class room (*n* = 20) (in percentages)	.26	.27
I always have to adapt tasks to the student with ASC's abilities in the classroom (*n* = 19)	−.05	.84
I never have to adapt tasks to the student with ASC's abilities in the classroom (*n* = 19)	−.16	.53
Specific activities to enhance social relations for the student with ASC (*n* = 18)	.09	.73
Specific activities to improve the attitudes of classmates towards the student with ASC (*n* = 18)	−.03	.92

**Table 5:** 

Statements	Likert scale steps	% disagree	% near agree	% agree	Weighted Kappa
I go on outings when the school arranges it	5	4.8	23.8	71.4	N.A.
My teacher helps me so I can concentrate during lesions	5	5.0	65.0	30.0	N.A.
I want to participate in P.E.	5	5.3	31.6	63.2	0.64
I talk to my classmates on the phone/internet after school	4	5.5	33.3	61.1	N.A.
It is hard for me to get friends	4	5.9	52.9	41.2	N.A.
I meet my classmates after school	4	9.1	50.0	40.9	0.13
I participate in P.E.	5	9.5	19.0	71.4	0.51
I want to go on outings when the school arranges it	5	10.0	35.0	55.0	N.A.
I talk to and are together with friends my age	5	10.5	42.1	47.4	0.31
I can get angry at someone that I like	4	11.1	61.1	27.8	0.02
Me and my classmates decide together what to do	4	13.6	77.3	9.1	0.05
My classmates likes me	4	14.3	28.6	12.0	0.37
I do what my classmates want to	4	15.0	25.0	60.0	0.15
I get help from my classmates in school	5	19.0	52.4	28.6	N.A.
My teacher is interested and ask me about what I am doing	5	20.0	50.0	30.0	0.28
I answer when my classmates talk to me	4	21.1	31.6	47.4	N.A.
I can ask for help if I hurt myself during school	4	21.1	47.4	31.6	0.06
I talk to people that are new to me	4	22.2	44.4	33.3	N.A.
I want my classmates to ask me if I want to join in with them	5	22.2	38.9	38.9	N.A.
I am with my classmates during recess	5	23.8	42.9	33.3	0.27
I decide myself what to do during recess	4	23.8	38.1	38.1	0.02
I am good at cooperating	4	25.0	45.0	30.0	N.A.
I sometimes pretend to like thinks I do not, for my classmates to like me	4	25.0	35.0	40.0	N.A.
I want to ask my classmates if I can join in with them	5	28.6	42.9	28.6	0.02
I want to be alone during recess	5	28.6	33.3	38.1	0.32
I want to answer my classmates when they talk to me	5	28.6	47.6	23.8	N.A.
I want to be with classmates during recess	5	28.6	47.6	23.8	0.17
I can understand when my teacher explains to the class what to do	5	30.0	45.0	25.0	0.09
I try again if I am unsuccessful	4	30.0	45.0	25.0	N.A.
I am afraid to make mistakes	4	30.0	40.0	30.0	0.09
It is easy for me to get friends	4	30.0	45.0	25.0	N.A.
I help my classmates in school	5	38.1	38.1	23.8	0.13
My classmates ask me if I want to join in with them	5	38.1	33.3	28.6	0.05
I ask my classmates if I can join in with them	5	38.1	38.1	23.8	0.21
I talk to my classmates in the classroom	5	38.1	42.9	19.0	0.09
I tell my classmates if I think they act badly towards me	4	38.1	38.1	23.8	0.06
I want to talk to classmates in the class room	5	38.1	38.1	23.8	0.25
I can talk to my teacher when I want something	5	38.1	19.0	42.9	N.A.
I think my teacher and I understand each other	5	38.1	52.4	9.5	N.A.
I know what I am good at	4	38.9	44.4	16.7	N.A.
I want to help my classmates during school	5	40.0	50.0	10.0	0.13
I talk to teachers during recess	5	40.0	45.0	15.0	0.00
I am bullied at school	5	42.1	26.3	31.6	N.A.
I work with different learning materials than my classmates	5	42.9	38.1	19.0	N.A.
Me and my classmates are together as long as I want to	5	45.0	35.0	20.0	0.03
I can like a friend even if we sometimes disagree	4	57.1	19.0	23.8	0.25

## Appendix

See Table 5.
